# Stimulation frequency determines the distribution of language positive cortical regions during navigated transcranial magnetic brain stimulation

**DOI:** 10.1186/s12868-015-0143-9

**Published:** 2015-02-18

**Authors:** Theresa Hauck, Noriko Tanigawa, Monika Probst, Afra Wohlschlaeger, Sebastian Ille, Nico Sollmann, Stefanie Maurer, Claus Zimmer, Florian Ringel, Bernhard Meyer, Sandro M Krieg

**Affiliations:** Department of Neurosurgery, Klinikum rechts der Isar, Technische Universität München, Ismaninger Str. 22, 81675 Munich, Germany; Faculty of Linguistics, Philology, & Phonetics, University of Oxford, Walton Street, Oxford, OX1 2HG UK; Section of Neuroradiology, Department of Radiology, Klinikum rechts der Isar, Technische Universität München, Ismaninger Str. 22, 81675 Munich, Germany

**Keywords:** Action naming, Cortical mapping, Frequency, Mapping protocol, Navigated brain stimulation, Object naming, Pseudoword reading, Transcranial magnetic stimulation, Verb generation

## Abstract

**Background:**

Although language mapping by repetitive navigated transcranial magnetic stimulation (rTMS) gains importance in neuropsychological research and clinical utility, neuroscientists still use different mapping protocols including different stimulation frequencies. To refine the existing language protocol, we tested two different repetition rates of 5 Hz/10 pulses and 7 Hz/10 pulses with a 0 ms delay in 19 healthy subjects. We furthermore investigated differences between both frequencies in case of performance of four different language tasks: object naming, pseudoword reading, verb generation, and action naming.

**Results:**

Even the small variance in frequencies revealed statistically significant differences concerning the number and type of language errors. Stimulation with 5 Hz evoked a higher number of all occurred language errors in all language tasks (error rate object naming 14% (5 Hz) vs. 12% (7 Hz); pseudoword reading 4% (5 Hz) vs. 3% (7 Hz); verb generation 13% (5 Hz) vs. 11% (7 Hz); action naming 11% (5 Hz) vs. 9% (7 Hz)), whereas 7 Hz evoked specifically more total speech arrests.

**Conclusion:**

These findings suggest that the stimulation frequency has to be adapted to the aim of the rTMS language investigation.

## Background

In 1991, Pascual-Leone et al. performed the first repetitive transcranial magnetic stimulation (TMS) language study in order to find a noninvasive method to determine the language-dominant hemisphere [1]. Thereby, repetitive TMS-induced language interruption could be observed, which was explained by causing a transient functional under-activity during stimulation: a ‘virtual lesion’. Shortly afterwards, a large number of repetitive TMS studies followed to examine basically the potentials and the reliability of this technique for clinical and research applications [2-4]. Thereby, several protocols have been tested by using different stimulus parameters, which has probably contributed to the high inter-study variability in occurrence of language errors. One of the parameters that have been particularly tested was the stimulus frequency. Initial TMS language studies used high frequencies up to 32 Hz and trains of up to 10 s (e.g., [1,2,4]), which resulted in discomfort. However, Epstein et al. found stronger speech arrest effects in combination with higher intensities, but higher speech arrest reliability when applying lower frequencies [4]. Furthermore, because higher frequencies led to more discomfort due to increased muscle stimulation, Epstein et al. suggested 4-8 Hz to be the best ratio of efficacy to pain.

In the last years, with the development of repetitive navigated TMS (rTMS), we now have the opportunity to directly visualize the stimulated cortical sites on the individual subject’s cranial MRI. This progress raises expectations concerning the accuracy and repeatability of the detection of language-positive sites, which is evident for clinical application. However, neuroscientists still have to choose appropriate stimulation parameters such as stimulation frequency, number of pulses, onset time, and stimulus intensity, so the question of the optimum language mapping protocol still remains unsolved.

Concerning the frequency, Epstein et al.’s findings are still accepted and lower frequency rTMS is widely used. Yet, today’s research groups use differing lower frequency rates, and also nowadays no definite stimulus protocol exists (e.g., [5-7]). In addition, accuracy and also number and location of language-related regions vary across rTMS language studies (e.g., [8,9]).

Thus, the present study compares rTMS language mapping at a frequency of 5 and 7 Hz. Four different language tasks were tested to examine validity regarding different language sub-functions in order to contribute to further refinement of the rTMS language protocol.

## Methods

### Study subjects

Twenty volunteers (10 female, 10 male) without any neurological disorders at the age of 24.6 ± 1.7 years (range 22–29; Table 1) were enrolled. All subjects were right-handed, which was assessed by the Edinburgh handedness inventory (84 ± 13 points) [10], and native German speakers without any additional mother tongue. In general, participation was only permitted at an age of at least 18 years and with the volunteers’ signed written informed consent. Exclusion criteria were bilateral or left-handedness, second mother tongue, previous seizures, pathological findings on cranial MRI, aberrant medical history, developmental language deficits, any neurological impairment, and general TMS exclusion criteria like pacemaker, deep brain stimulation, or cochlear implant [11].

**Table 1:** **Pain, motor threshold (MT) intensity, and baseline performance**

		**5 Hz/10 pulses**	**7 Hz/10 pulses**	**p**
		**Mean ± SD**		
Pain (VAS)	Convexity	1.7 ± 1.6	2.2 ± 1.6	p = 0.432
	Temporal	5.4 ± 2.2	5.0 ± 2.0	p = 0.540
Motor threshold intensity (% stimulator output)		33.1 ± 4.8	33.5 ± 5.1	p = 0.796
Representative correct baseline pictures (out of a dataset of 100 items)	Object naming	92.3 ± 3.4		p < 0.0001
	Pseudoword reading	96.0 ± 2.2		
	Verb generation	89.5 ± 4.3		
	Action naming	89.2 ± 5.6		

Stimulation parameters used in the study, including pain score according to the visual analogue scale (VAS); RMT = resting motor threshold (% stimulator output); and representative correct baseline pictures. Data is presented for a stimulation train frequency of 5 Hz and of 7 Hz and as mean ± standard deviation (SD).

### Study design

Two mapping sessions were performed within 13 to 15 days. One session was performed with a frequency of 5 Hz, the other session was performed with 7 Hz; in each session we tested four different language tasks. Hence, we examined the impact of different mapping frequencies on the error rate and error location. The subjects underwent the investigation in a random order.

### Ethics

The experimental procedures were approved by the local ethical committee of the Technische Universität München (TUM) in accordance with the declaration of Helsinki (registration number: 2793/10). Prior to the first language-mapping session, all participants provided written informed consent.

### Navigational MRI scan

Prior to rTMS language mapping, all participants received a navigational MRI for neuronavigation. It was performed on a 3 Tesla MR scanner (Achieva 3T, Philips Medical Systems, The Netherlands B.V.) with an 8-channel phased array head coil. A 3D fast field echo sequence (TR/TE 8.3/3.9 ms, 1mm^3^ isovoxel covering the whole head, 5 minutes 56 seconds acquisition time) without intravenous contrast administration was used for anatomical co-registration before the 3D dataset was transferred to the nTMS system via DICOM standard.

### rTMS language mapping

#### Experimental setup

The subjects underwent rTMS language mapping of the left hemisphere two times. Both mappings were performed with the Nexstim eXimia NBS system 4.3 and a NexSpeech® module (Nexstim Oy, Helsinki, Finland).

To prevent inter-observer variability, the first author performed both mapping sessions. In the naming and generation tasks, we used the same task items during the first and second mapping. Thus, we set a time span of thirteen to fifteen days between the two examinations to minimize training effects.

Each mapping session followed the same protocol as described previously [8,12], yet we used a picture-to-trigger interval of 0 instead of 300 ms [9]. Briefly, the examination commenced determining the Resting Motor Threshold (RMT) by motor mapping of the cortical representation of the left-sided hand area (abductor pollicis brevis muscle and abductor digiti minimi muscle), as published earlier [13]. The location of the hand knob was identified prior to the first investigation and was saved for the second one, where only the threshold had to be determined again. The stimulus intensity was adjusted to the RMT [8,12,14]. Deviating from the above-mentioned earlier protocol, as there was no necessity of increasing the RMT due to lacking language disruption or decreasing the RMT due to inaccaptable pain, we strictly applied 100% RMT as intensity during the language-mapping procedure.

In both sessions trains of 10 bursts were applied via rTMS; one investigation was performed with 5 Hz, the other one with 7 Hz.

#### Language tasks

We used four language tasks consisting of a set of 100 items each: object naming, pseudoword reading, verb generation, and action naming. All tasks had to be performed in German. Pictures and words were randomly displayed on a screen 60 cm in front of the subject.

The object naming task consisted of colored pictures of common objects which had to be named without article. The objects were similar to those listed in the established Snodgrass and Vanderwart picture set (1980) [8,15].

For the pseudoword reading task, we used items of a German word list by Felty et al. [16], which contained disyllabic nouns, verbs, and adjectives in the CVCCVC structure (C = Consonant, V = Vowel). Felty et al. derived pseudowords from these words. The subjects had to read aloud 50 pseudowords randomly mixed with 50 real words that served as a control. For the second mapping session, we used different pseudowords and real words, but from the same word list in order to minimize the learning effect.

The verb generation task was also a visual task: on a screen we demonstrated pictures of common objects out of which the subjects had to build verbs.

For the action naming task, the participants had to name pictures that showed daily activities (e.g., walking, sleeping, dancing) without using a noun.

#### Language-mapping procedure

Prior to each language task, baseline testing was performed. Thereby, all randomly displayed pictures or words had to be named or read quickly and clearly. To consider variances in the subjects’ language lexica, misnamed or misread items were rejected from the stimulus sequence and representative correct baseline pictures were documented (Table 1). Only when the baseline testing of one task and the corresponding performance of that task during stimulation was terminated did we move on with the next language task.

As some of the pseudowords of the pseudoword reading task were quite long, they were displayed for 1.0 s. For pictures of the object naming, action naming, and verb generation task, we used a display time of 0.7 s. The inter-picture interval (IPI) of all tasks was 3.0 s. After baseline testing, the actual stimulation progress followed with the same display times and IPI. The PTI was 0 ms, thus magnetic pulses were applied simultaneously with the item presentation.

Pain during stimulation was measured with the visual analogue scale (VAS) (Table 1). Altogether, each of the two language mapping sessions required about 90 - 120 min per participant.

For objective and detailed language analysis, video recording of baseline performance as well as of language mapping was conducted [7].

#### Stimulated points

For a description of brain areas, we use the cortical parcellation system (CPS; Table 2) based on Corina et al. [17].

**Table 2:** **Anatomical names and abbreviations of the cortical parcellation system (CPS)**

**Abbreviation**	**Anatomy**
anG	angular gyrus
aSMG	anterior supramarginal gyrus
aSTG	anterior superior temporal gyrus
dPoG	dorsal post-central gyrus
dPrG	dorsal pre-central gyrus
mMFG	middle middle frontal gyrus
mMTG	middle middle temporal gyrus
mPoG	middle post-central gyrus
mPrG	middle pre-central gyrus
mSFG	middle superior frontal gyrus
mSTG	middle superior temporal gyrus
opIFG	opercular inferior frontal gyrus
orIFG	orbital part of the inferior frontal gyrus
pMFG	posterior middle frontal gyrus
pMTG	posterior middle temporal gyrus
polIFG	polar inferior frontal gyrus
polMFG	polar middle frontal gyrus
polMTG	polar middle temporal gyrus
polSFG	polar superior frontal gyrus
polSTG	polar superior temporal gyrus
pSFG	posterior superior frontal gyrus
pSMG	posterior supramarginal gyrus
pSTG	posterior superior temporal gyrus
SPL	superior parietal lobe
trIFG	triangular inferior frontal gyrus
vPoG	ventral post-central gyrus
vPrG	ventral pre-central gyrus

Anatomical names and abbreviations of the cortical parcellation system (CPS) according to Corina et al. [17].

**Figure 1:**
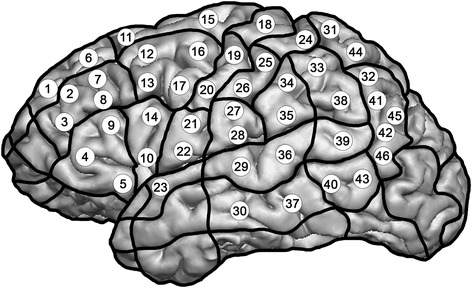
**Outline of the 46 stimulated cortical spots.** In each task, each point was stimulated three times.

Figure 1 shows the 46 previously determined cortical spots, which were defined anatomically and stimulated in each mapping session. Each of the 46 points was stimulated three times per language task, which equals 138 stimulation trains per task. Prior to the first investigation, the spots were anatomically identified and tagged on the 3D MRI. They could be saved for the second mapping session, so we stimulated exactly the same points during both investigations. Regions which were not stimulated by rTMS were the orbital part of the inferior frontal gyrus (orIFG), polar superior and polar middle temporal gyrus (polSTG, polMTG), anterior middle temporal gyrus (aMTG) and polar superior, polar middle, and polar inferior frontal gyrus (polSFG, polMFG, polIFG). Those regions were not stimulated because stimulation of these regions causes inacceptable pain, as published earlier [18]. The extension of stimulated areas had to be furthermore restricted due to increasing distance between skin and brain. At the inferior temporal gyrus (ITG), stimulation intensity decreased below 50 V/m. Thus, ITG was also not mapped, as outlined in previous rTMS reports [18].

During stimulation, the coil was moved tangentially to the skull in a strict anterior-posterior field orientation [4,7,19]. The field strength at the region of interest ranged between 55-80 V/m; minimum field strength was 55 V/m.

### Mapping session analysis

The recorded videos of all mapping sessions were analyzed, as described in earlier publications [7,8,12], blinded to stimulated cortical spots and previous results. For the analysis, baseline naming was compared to naming performance during rTMS. Any site at which stimulation evoked language impairment was marked as a language-related site. Thereby, we divided the evoked errors into different categories: No response errors, performance errors, hesitations, neologisms, semantic paraphasias, and phonological paraphasias as outlined in previous studies [20,21]. Furthermore, verb generation and action naming also included nominalization errors defined as an inability to name the appropriate verb, in which case the noun was named instead. We summarized all types of errors in the error category “all errors”. Errors attributed to muscle stimulation or discomfort were discarded from further analysis; thereby we paid attention to any expression of the participants’ faces with discomfort during video analysis. One spot was considered as language positive if at least one out of the three stimulations per spot caused any error.

To compare the two frequencies, we calculated error rates for each stimulation point defined as the number of elicited language errors at each stimulated site per total number of stimulations of this site. Error rates are presented as percentage.

### Statistics

Pain (VAS) and RMT were presented as mean ± standard deviation (SD). We tested differences of those parameters between the first and the second mapping by using the Wilcoxon matched-pairs signed rank test. For testing differences of baseline performance between the four tasks, we performed Friedman’s test for non-parametric matched groups.

The Wilcoxon matched-pairs signed rank test was used again for each language task for testing differences among distribution of error rates per stimulation point in different mapping frequencies. A value of p < 0.05 was considered significant (GraphPad Prism 6.0, La Jolla, CA, USA).

## Results

### Stimulation-related incidents and discomfort

Nineteen out of the 20 included participants tolerated the stimulation well. However, one subject complained about intensive discomfort and developed vegetative symptoms including perspiration and nausea during RMT determination prior to rTMS. In that case, we stopped the investigation in this subject.

Maximum pain according to the VAS was comparable in the first and the second mapping (Table 1).

### Baseline and rTMS mapping parameters

RMT intensities in both examinations showed no significant difference (p = 0.796; Table 1). Differences among the number of correctly named baseline pictures were statistically significant (p < 0.0001; Table 1).

### Language errors induced by different frequencies

#### Sum of all errors

**Figure 2:**
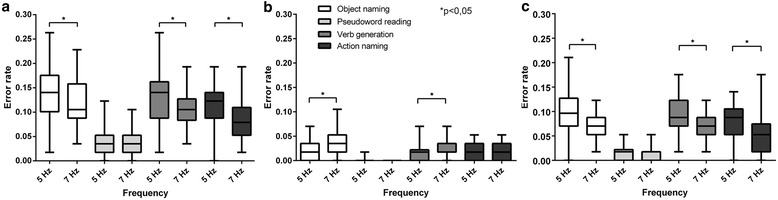
**Overall error rates (a), no response error rates (b), and hesitation error rates (c) revealed by language mapping via rTMS.** Distribution of elicited naming errors while performing object naming, pseudoword reading, verb generation, and action naming shown for stimulation with 5 Hz and 7 Hz.

When taking into account all error categories, during all four language tasks our data showed higher error rates at a repetition rate of 5 Hz than at 7 Hz repetition rate (Figure 2a; Table 3).

**Table 3:** **Summary of naming errors induced by rTMS**

***Error category***	**Object naming**			**Pseudoword reading**			**Verb generation**			**Action naming**		
	**5 Hz**	**7 Hz**	**p**	**5 Hz**	**7 Hz**	**p**	**5 Hz**	**7 Hz**	**p**	**5 Hz**	**7 Hz**	**p**
**No response**	1.6%	3.5%	0.001	0.0%	0.0%	>0.999	1.6%	2.4%	0.015	1.8%	1.9%	0.908
**Hesitations**	9.9%	7.2%	0.002	1.5%	1.1%	0.213	9.5%	6.7%	<0.001	7.8%	5.5%	0.004
**Performance**	1.3%	0.8%	0.112	0.8%	0.8%	0.952	1.0%	0.6%	0.307	0.6%	0.5%	0.692
**Phonological**	0.2%	0.0%	0.125	1.6%	1.4%	0.435	0.4%	0.2%	0.075	0.4%	0.3%	0.677
**Semantic**	0.6%	0.4%	0.357	0.0%	0.0%	--	0.3%	0.3%	>0.999	0.3%	0.2%	0.774
**Neologism**	0.0%	0.0%	>0.999	0.0%	0.0%	>0.999	0.1%	0.0%	0.250	0.0%	0.0%	>0.999
**Nominalization**	0.0%	0.0%	--	0.0%	0.0%	--	0.6%	0.3%	0.364	0.2%	0.2%	>0.999
**All errors**	13.7%	11.5%	0.021	4.0%	3.2%	0.202	13.5%	10.6%	0.002	11.1%	8.5%	0.005

Naming error rates of each error type in four tested language tasks and for both applied frequencies. Differences among distribution of error rates per stimulation point in different mapping frequencies were tested by the Wilcoxon matched-pairs signed rank test; respective p values were presented.

**Figure 3:**
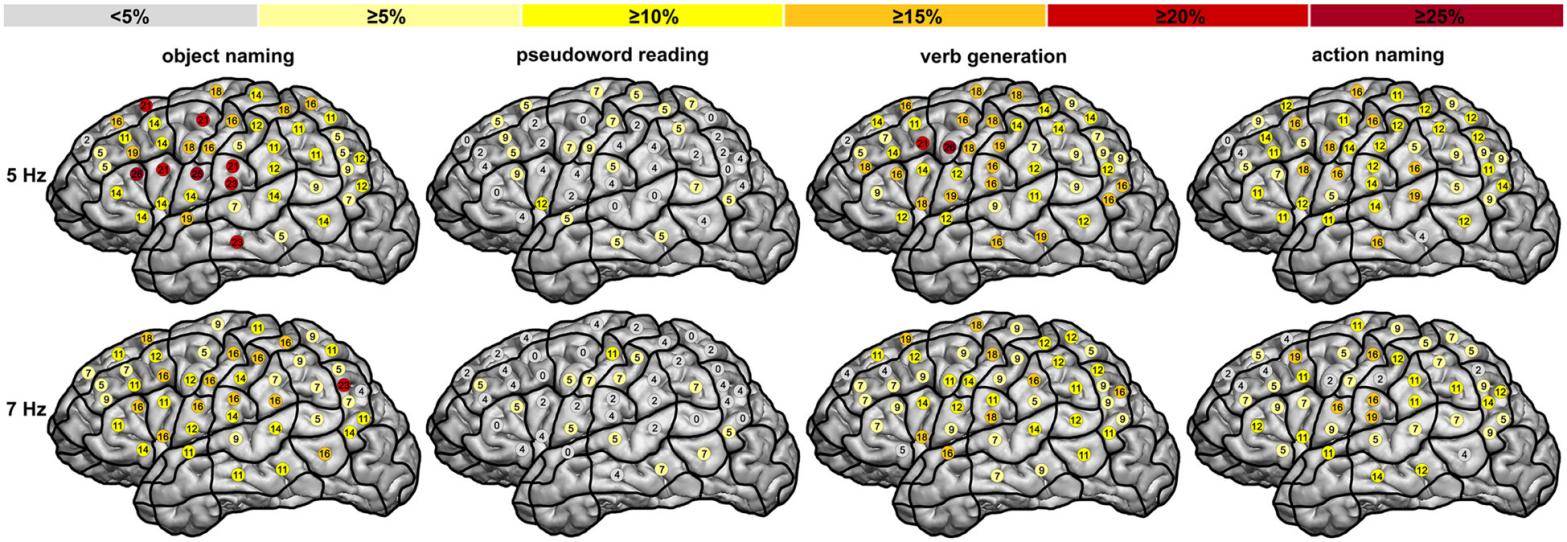
**Distribution of all elicited naming errors (error rates) while task performing.** Results are demonstrated for the object naming, pseudoword reading, verb generation, and action naming task for 5 Hz and for 7 Hz.

The distribution of language-positive regions showed statistically significant differences between 5 Hz and 7 Hz during object naming, verb generation, and action naming; pseudoword reading revealed no distinct differences (Figure 2a; Table 3). The exact appearance of errors during stimulation is visualized in Figure 3 and Table 4.

**Table 4:** **Summary of all naming errors induced by rTMS trains**

	**Object naming**		**Pseudoword reading**		**Verb generation**		**Action naming**	
**Stimulation point**	***5 Hz***	***7 Hz***	***5 Hz***	***7 Hz***	***5 Hz***	***7 Hz***	***5 Hz***	***7 Hz***
1	1.8%	7.0%	0.0%	1.8%	1.8%	3.5%	0.0%	1.8%
2	5.3%	5.3%	1.8%	5.3%	5.3%	7.0%	3.5%	3.5%
3	5.3%	8.8%	3.5%	3.5%	17.5%	7.0%	5.3%	7.0%
4	14.0%	10.5%	0.0%	0.0%	8.8%	7.0%	10.5%	12.3%
5	14.0%	14.0%	3.5%	3.5%	12.3%	5.3%	10.5%	5.3%
6	15.8%	10.5%	5.3%	1.8%	14.0%	10.5%	8.8%	5.3%
7	10.5%	7.0%	8.8%	3.5%	7.0%	3.5%	14.0%	3.5%
8	19.3%	10.5%	5.3%	3.5%	14.0%	7.0%	10.5%	5.3%
9	26.3%	15.8%	8.8%	5.3%	15.8%	8.8%	7.0%	8.8%
10	14.0%	15.8%	12.3%	3.5%	17.5%	17.5%	12.3%	10.5%
11	21.1%	17.5%	5.3%	3.5%	15.8%	19.3%	12.3%	3.5%
12	14.0%	12.3%	1.8%	0.0%	10.5%	12.3%	15.8%	19.3%
13	14.0%	15.8%	1.8%	0.0%	21.1%	8.8%	5.3%	10.5%
14	21.1%	10.5%	7.0%	1.8%	14.0%	14.0%	17.5%	7.0%
15	17.5%	8.8%	3.5%	3.5%	17.5%	17.5%	15.8%	10.5%
16	21.1%	5.3%	0.0%	0.0%	15.8%	8.8%	10.5%	5.3%
17	17.5%	12.3%	7.0%	5.3%	26.3%	10.5%	17.5%	1.8%
18	14.0%	10.5%	5.3%	1.8%	17.5%	8.8%	10.5%	8.8%
19	15.8%	15.8%	7.0%	10.5%	17.5%	17.5%	15.8%	15.8%
20	15.8%	15.8%	8.8%	7.0%	17.5%	14.0%	14.0%	7.0%
21	24.6%	15.8%	3.5%	1.8%	12.3%	12.3%	15.8%	15.8%
22	14.0%	12.3%	1.8%	5.3%	19.3%	8.8%	5.3%	8.8%
23	19.3%	10.5%	5.3%	0.0%	12.3%	15.8%	10.5%	10.5%
24	17.5%	15.8%	5.3%	3.5%	14.0%	12.3%	12.3%	5.3%
25	12.3%	15.8%	1.8%	5.3%	14.0%	8.8%	12.3%	12.3%
26	5.3%	14.0%	3.5%	7.0%	19.3%	8.8%	12.3%	1.8%
27	21.1%	15.8%	5.3%	1.8%	15.8%	10.5%	12.3%	15.8%
28	22.8%	14.0%	3.5%	3.5%	15.8%	17.5%	14.0%	19.3%
29	7.0%	8.8%	0.0%	5.3%	8.8%	7.0%	14.0%	5.3%
30	22.8%	10.5%	5.3%	3.5%	15.8%	7.0%	15.8%	14.0%
31	15.8%	8.8%	7.0%	0.0%	8.8%	12.3%	8.8%	7.0%
32	5.3%	5.3%	1.8%	0.0%	7.0%	12.3%	12.3%	1.8%
33	10.5%	8.8%	5.3%	1.8%	14.0%	12.3%	12.3%	7.0%
34	10.5%	7.0%	3.5%	3.5%	7.0%	15.8%	5.3%	10.5%
35	12.3%	15.8%	3.5%	1.8%	12.3%	5.3%	15.8%	10.5%
36	14.0%	14.0%	0.0%	1.8%	10.5%	14.0%	19.3%	7.0%
37	5.3%	10.5%	5.3%	7.0%	19.3%	8.8%	3.5%	12.3%
38	10.5%	7.0%	1.8%	7.0%	8.8%	10.5%	8.8%	10.5%
39	8.8%	5.3%	7.0%	0.0%	8.8%	12.3%	5.3%	7.0%
40	14.0%	15.8%	3.5%	7.0%	12.3%	10.5%	12.3%	3.5%
41	5.3%	22.8%	1.8%	3.5%	8.8%	8.8%	8.8%	8.8%
42	8.8%	7.0%	0.0%	3.5%	12.3%	8.8%	10.5%	14.0%
43	7.0%	14.0%	5.3%	5.3%	15.8%	10.5%	8.8%	8.8%
44	10.5%	10.5%	0.0%	1.8%	14.0%	5.3%	12.3%	5.3%
45	12.3%	3.5%	3.5%	3.5%	8.8%	15.8%	8.8%	12.3%
46	12.3%	10.5%	3.5%	0.0%	15.8%	8.8%	14.0%	5.3%
total	13.7%	11.5%	4.0%	3.2%	13.5%	10.6%	11.1%	8.5%

Summary of all naming errors induced by rTMS trains. Results are demonstrated as error rates per stimulation point and as the sum of all stimulation points for each language task.

#### No response errors

**Figure 4:**
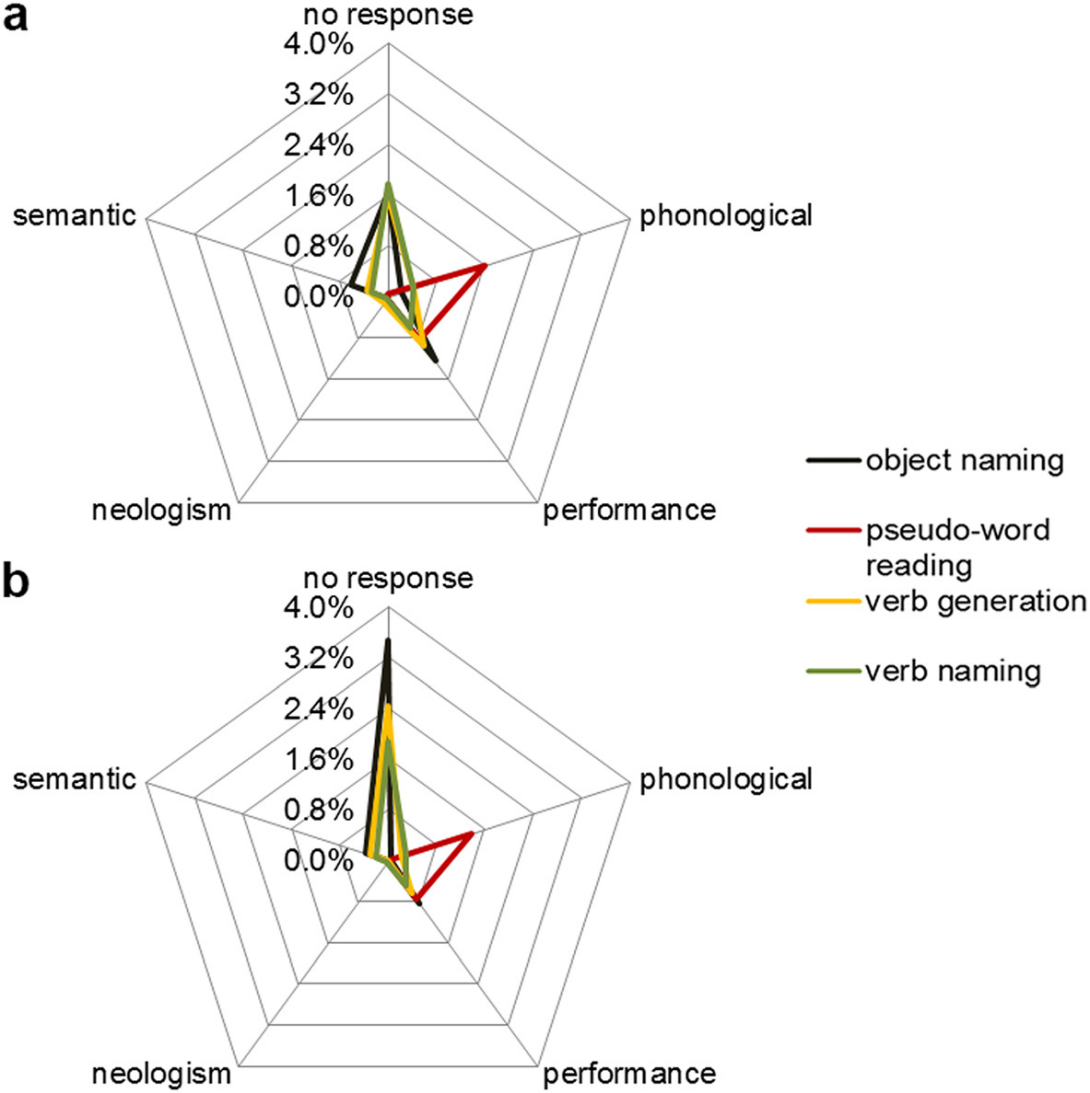
**Error type (%) differences across task types.** Error rates per error category during object naming, pseudoword reading, verb generation, and action naming shown for 5 Hz **(a)** and 7 Hz **(b)**. For detailed values see also Table 3.

During both frequencies, no response errors formed after hesitations the second most-frequent error category in object naming, verb generation and action naming (Table 3). In the reading task, no response errors appeared less frequently than phonological errors and hesitations (Table 3). Comparing 5 Hz with 7 Hz, in this error category we observed higher error rates at a repetition rate of 7 Hz (Figures 2b and 4a and b; Table 3). Those differences were statistically significant during object naming and verb generation, but not during pseudoword reading and action naming (Table 3). Table 5 shows the error rates per stimulation point for all tasks and for both frequencies.

**Table 5:** **Summary of all no response errors induced by rTMS trains**

	**Object naming**		**Pseudoword reading**		**Verb generation**		**Action naming**	
**Stimulation point**	***5 Hz***	***7 Hz***	***5 Hz***	***7 Hz***	***5 Hz***	***7 Hz***	***5 Hz***	***7 Hz***
1	0.0%	0.0%	0.0%	0.0%	0.0%	0.0%	0.0%	0.0%
2	0.0%	0.0%	0.0%	0.0%	1.8%	5.3%	0.0%	0.0%
3	1.8%	3.5%	0.0%	0.0%	0.0%	1.8%	0.0%	0.0%
4	7.0%	1.8%	0.0%	0.0%	1.8%	1.8%	1.8%	1.8%
5	0.0%	3.5%	0.0%	0.0%	0.0%	3.5%	0.0%	1.8%
6	3.5%	0.0%	0.0%	0.0%	0.0%	0.0%	0.0%	0.0%
7	0.0%	0.0%	0.0%	0.0%	3.5%	0.0%	0.0%	0.0%
8	3.5%	7.0%	0.0%	0.0%	1.8%	1.8%	0.0%	0.0%
9	5.3%	3.5%	0.0%	0.0%	0.0%	3.5%	0.0%	0.0%
10	3.5%	3.5%	0.0%	0.0%	0.0%	3.5%	1.8%	1.8%
11	0.0%	7.0%	0.0%	0.0%	5.3%	3.5%	1.8%	0.0%
12	0.0%	5.3%	0.0%	0.0%	3.5%	7.0%	3.5%	3.5%
13	0.0%	10.5%	0.0%	0.0%	0.0%	0.0%	1.8%	3.5%
14	0.0%	5.3%	0.0%	0.0%	0.0%	1.8%	0.0%	1.8%
15	3.5%	1.8%	0.0%	0.0%	0.0%	5.3%	1.8%	0.0%
16	3.5%	0.0%	0.0%	0.0%	1.8%	1.8%	5.3%	0.0%
17	1.8%	0.0%	0.0%	0.0%	1.8%	1.8%	1.8%	0.0%
18	0.0%	1.8%	0.0%	0.0%	1.8%	0.0%	0.0%	1.8%
19	3.5%	10.5%	1.8%	0.0%	1.8%	7.0%	0.0%	3.5%
20	0.0%	5.3%	0.0%	0.0%	3.5%	1.8%	1.8%	1.8%
21	5.3%	8.8%	0.0%	0.0%	0.0%	3.5%	1.8%	5.3%
22	0.0%	7.0%	0.0%	0.0%	1.8%	3.5%	1.8%	3.5%
23	3.5%	0.0%	0.0%	0.0%	0.0%	1.8%	0.0%	1.8%
24	0.0%	5.3%	0.0%	0.0%	1.8%	1.8%	5.3%	1.8%
25	0.0%	3.5%	0.0%	0.0%	1.8%	5.3%	0.0%	5.3%
26	0.0%	5.3%	0.0%	0.0%	1.8%	0.0%	3.5%	0.0%
27	1.8%	3.5%	0.0%	0.0%	3.5%	1.8%	5.3%	5.3%
28	3.5%	3.5%	0.0%	0.0%	3.5%	1.8%	1.8%	1.8%
29	0.0%	1.8%	0.0%	0.0%	1.8%	1.8%	0.0%	1.8%
30	1.8%	1.8%	0.0%	0.0%	7.0%	1.8%	5.3%	1.8%
31	1.8%	5.3%	0.0%	0.0%	1.8%	0.0%	1.8%	0.0%
32	0.0%	3.5%	0.0%	0.0%	0.0%	3.5%	3.5%	0.0%
33	0.0%	0.0%	0.0%	0.0%	1.8%	3.5%	0.0%	1.8%
34	0.0%	0.0%	0.0%	0.0%	1.8%	3.5%	0.0%	1.8%
35	0.0%	7.0%	0.0%	0.0%	1.8%	1.8%	5.3%	5.3%
36	3.5%	1.8%	0.0%	0.0%	0.0%	3.5%	5.3%	5.3%
37	5.3%	1.8%	0.0%	0.0%	3.5%	1.8%	1.8%	3.5%
38	1.8%	1.8%	0.0%	0.0%	0.0%	3.5%	1.8%	0.0%
39	0.0%	3.5%	0.0%	0.0%	3.5%	5.3%	0.0%	5.3%
40	1.8%	3.5%	0.0%	0.0%	0.0%	1.8%	1.8%	0.0%
41	0.0%	8.8%	0.0%	0.0%	0.0%	1.8%	3.5%	1.8%
42	0.0%	0.0%	0.0%	0.0%	1.8%	0.0%	1.8%	1.8%
43	1.8%	5.3%	0.0%	0.0%	0.0%	1.8%	0.0%	1.8%
44	1.8%	1.8%	0.0%	0.0%	3.5%	1.8%	3.5%	0.0%
45	1.8%	1.8%	0.0%	0.0%	3.5%	3.5%	5.3%	3.5%
46	3.5%	3.5%	0.0%	0.0%	0.0%	1.8%	1.8%	5.3%
total	1.6%	3.5%	0.0%	0.0%	1.6%	2.4%	1.8%	1.9%

Summary of all no response errors induced by rTMS trains. Results are demonstrated as error rates per stimulation point and as the sum of all stimulation points for each language task.

#### Hesitations

Hesitations were the most frequently observed errors during object naming, verb generation and action naming (Table 3). During pseudoword reading, only phonological errors appeared more often (Table 3). Furthermore, our results consistently showed more hesitations when stimulating with 5 Hz than with 7 Hz (Figure 2c). The difference in the distribution of errors was statistically significant, except for the pseudoword reading task (Table 3). Hesitation error rates per stimulation point are presented in detail by Table 6.

**Table 6:** **Summary of all hesitations induced by rTMS trains**

	**Object naming**		**Pseudoword reading**		**Verb generation**		**Action naming**	
**Stimulation point**	***5 Hz***	***7 Hz***	***5 Hz***	***7 Hz***	***5 Hz***	***7 Hz***	***5 Hz***	***7 Hz***
1	1.8%	5.3%	0.0%	1.8%	1.8%	3.5%	0.0%	1.8%
2	5.3%	5.3%	1.8%	1.8%	3.5%	1.8%	1.8%	1.8%
3	1.8%	5.3%	1.8%	1.8%	15.8%	5.3%	5.3%	7.0%
4	7.0%	7.0%	0.0%	0.0%	5.3%	3.5%	7.0%	8.8%
5	14.0%	8.8%	0.0%	0.0%	10.5%	1.8%	10.5%	1.8%
6	8.8%	10.5%	3.5%	0.0%	12.3%	5.3%	8.8%	3.5%
7	8.8%	7.0%	5.3%	1.8%	1.8%	3.5%	8.8%	1.8%
8	14.0%	3.5%	3.5%	0.0%	8.8%	5.3%	8.8%	5.3%
9	10.5%	7.0%	5.3%	1.8%	12.3%	5.3%	7.0%	7.0%
10	8.8%	10.5%	1.8%	3.5%	12.3%	10.5%	8.8%	5.3%
11	21.1%	10.5%	3.5%	1.8%	8.8%	10.5%	10.5%	3.5%
12	7.0%	7.0%	0.0%	0.0%	7.0%	5.3%	7.0%	12.3%
13	12.3%	5.3%	0.0%	0.0%	17.5%	7.0%	3.5%	7.0%
14	17.5%	3.5%	1.8%	1.8%	8.8%	8.8%	8.8%	3.5%
15	7.0%	7.0%	0.0%	0.0%	10.5%	12.3%	10.5%	8.8%
16	15.8%	5.3%	0.0%	0.0%	14.0%	7.0%	5.3%	3.5%
17	12.3%	8.8%	0.0%	0.0%	12.3%	7.0%	14.0%	1.8%
18	14.0%	7.0%	5.3%	0.0%	12.3%	5.3%	8.8%	5.3%
19	10.5%	5.3%	5.3%	5.3%	14.0%	8.8%	12.3%	12.3%
20	10.5%	5.3%	0.0%	0.0%	14.0%	8.8%	10.5%	1.8%
21	17.5%	7.0%	0.0%	0.0%	8.8%	8.8%	14.0%	7.0%
22	10.5%	5.3%	0.0%	1.8%	14.0%	1.8%	3.5%	1.8%
23	10.5%	8.8%	1.8%	0.0%	7.0%	8.8%	10.5%	5.3%
24	17.5%	10.5%	0.0%	0.0%	10.5%	8.8%	5.3%	1.8%
25	10.5%	12.3%	1.8%	3.5%	8.8%	3.5%	10.5%	5.3%
26	5.3%	7.0%	0.0%	3.5%	15.8%	3.5%	5.3%	1.8%
27	15.8%	7.0%	1.8%	0.0%	12.3%	7.0%	3.5%	10.5%
28	19.3%	10.5%	0.0%	1.8%	8.8%	12.3%	12.3%	17.5%
29	3.5%	7.0%	0.0%	0.0%	5.3%	5.3%	12.3%	3.5%
30	19.3%	7.0%	3.5%	0.0%	8.8%	5.3%	10.5%	10.5%
31	12.3%	3.5%	3.5%	0.0%	3.5%	12.3%	7.0%	7.0%
32	1.8%	1.8%	1.8%	0.0%	5.3%	8.8%	5.3%	0.0%
33	10.5%	7.0%	1.8%	0.0%	10.5%	8.8%	12.3%	3.5%
34	7.0%	7.0%	3.5%	1.8%	5.3%	8.8%	5.3%	8.8%
35	10.5%	8.8%	1.8%	0.0%	8.8%	1.8%	7.0%	5.3%
36	7.0%	12.3%	0.0%	0.0%	10.5%	7.0%	10.5%	1.8%
37	0.0%	8.8%	0.0%	5.3%	8.8%	5.3%	1.8%	5.3%
38	7.0%	5.3%	0.0%	3.5%	7.0%	7.0%	5.3%	10.5%
39	7.0%	1.8%	0.0%	0.0%	3.5%	5.3%	5.3%	1.8%
40	10.5%	12.3%	0.0%	0.0%	10.5%	8.8%	10.5%	3.5%
41	5.3%	12.3%	1.8%	1.8%	8.8%	7.0%	5.3%	7.0%
42	8.8%	7.0%	0.0%	1.8%	7.0%	8.8%	8.8%	8.8%
43	5.3%	8.8%	1.8%	1.8%	14.0%	7.0%	8.8%	7.0%
44	8.8%	7.0%	0.0%	1.8%	8.8%	1.8%	7.0%	5.3%
45	7.0%	1.8%	3.5%	1.8%	5.3%	10.5%	1.8%	8.8%
46	7.0%	7.0%	1.8%	0.0%	14.0%	7.0%	10.5%	0.0%
total	9.9%	7.2%	1.5%	1.1%	9.5%	6.7%	7.8%	5.5%

Summary of all hesitations induced by rTMS trains. Results are demonstrated as error rates per stimulation point and as the sum of all stimulation points for each language task.

#### Performance errors

Except for the reading task, performance errors appeared slightly more often at a repetition rate of 5 Hz (Figure 4a and b; Table 3). In all four language tasks, differences between 5 Hz and 7 Hz concerning performance errors were not statistically significant (Table 3). Error rates per stimulation point for both frequencies and for all tasks are described in Table 7.

**Table 7:** **Summary of all performance errors induced by rTMS trains**

	**Object naming**		**Pseudoword reading**		**Verb generation**		**Action naming**	
**Stimulation point**	***5 Hz***	***7 Hz***	***5 Hz***	***7 Hz***	***5 Hz***	***7 Hz***	***5 Hz***	***7 Hz***
1	0.0%	1.8%	0.0%	0.0%	0.0%	0.0%	0.0%	0.0%
2	0.0%	0.0%	0.0%	0.0%	0.0%	0.0%	0.0%	1.8%
3	0.0%	0.0%	0.0%	0.0%	1.8%	0.0%	0.0%	0.0%
4	0.0%	1.8%	0.0%	0.0%	0.0%	1.8%	1.8%	0.0%
5	0.0%	1.8%	0.0%	0.0%	1.8%	0.0%	0.0%	1.8%
6	0.0%	0.0%	0.0%	1.8%	1.8%	0.0%	0.0%	1.8%
7	0.0%	0.0%	0.0%	0.0%	0.0%	0.0%	1.8%	0.0%
8	1.8%	0.0%	0.0%	3.5%	0.0%	0.0%	0.0%	0.0%
9	7.0%	5.3%	1.8%	0.0%	1.8%	0.0%	0.0%	1.8%
10	0.0%	1.8%	7.0%	0.0%	1.8%	1.8%	1.8%	3.5%
11	0.0%	0.0%	0.0%	0.0%	0.0%	1.8%	0.0%	0.0%
12	1.8%	0.0%	0.0%	0.0%	0.0%	0.0%	0.0%	0.0%
13	0.0%	0.0%	1.8%	0.0%	0.0%	1.8%	0.0%	0.0%
14	3.5%	1.8%	3.5%	0.0%	0.0%	0.0%	3.5%	0.0%
15	3.5%	0.0%	0.0%	1.8%	5.3%	0.0%	3.5%	1.8%
16	1.8%	0.0%	0.0%	0.0%	0.0%	0.0%	0.0%	0.0%
17	1.8%	3.5%	3.5%	5.3%	3.5%	1.8%	0.0%	0.0%
18	0.0%	1.8%	0.0%	0.0%	0.0%	1.8%	0.0%	0.0%
19	0.0%	0.0%	0.0%	0.0%	1.8%	0.0%	0.0%	0.0%
20	5.3%	3.5%	5.3%	3.5%	0.0%	1.8%	0.0%	1.8%
21	1.8%	0.0%	0.0%	0.0%	1.8%	0.0%	0.0%	1.8%
22	1.8%	0.0%	0.0%	0.0%	3.5%	1.8%	0.0%	1.8%
23	3.5%	1.8%	0.0%	0.0%	3.5%	0.0%	0.0%	1.8%
24	0.0%	0.0%	1.8%	0.0%	1.8%	0.0%	0.0%	1.8%
25	0.0%	0.0%	0.0%	1.8%	0.0%	0.0%	1.8%	0.0%
26	0.0%	1.8%	3.5%	1.8%	1.8%	3.5%	1.8%	0.0%
27	1.8%	5.3%	1.8%	0.0%	0.0%	1.8%	1.8%	0.0%
28	0.0%	0.0%	0.0%	0.0%	0.0%	3.5%	0.0%	0.0%
29	3.5%	0.0%	0.0%	1.8%	1.8%	0.0%	0.0%	0.0%
30	1.8%	1.8%	1.8%	1.8%	0.0%	0.0%	0.0%	0.0%
31	1.8%	0.0%	0.0%	0.0%	1.8%	0.0%	0.0%	0.0%
32	3.5%	0.0%	0.0%	0.0%	1.8%	0.0%	3.5%	1.8%
33	0.0%	1.8%	0.0%	1.8%	0.0%	0.0%	0.0%	0.0%
34	3.5%	0.0%	0.0%	1.8%	0.0%	3.5%	0.0%	0.0%
35	1.8%	0.0%	0.0%	0.0%	1.8%	0.0%	1.8%	0.0%
36	0.0%	0.0%	0.0%	0.0%	0.0%	1.8%	1.8%	0.0%
37	0.0%	0.0%	3.5%	1.8%	0.0%	0.0%	0.0%	0.0%
38	1.8%	0.0%	0.0%	0.0%	1.8%	0.0%	1.8%	0.0%
39	0.0%	0.0%	3.5%	0.0%	0.0%	0.0%	0.0%	0.0%
40	1.8%	0.0%	0.0%	0.0%	0.0%	0.0%	0.0%	0.0%
41	0.0%	1.8%	0.0%	1.8%	0.0%	0.0%	0.0%	0.0%
42	0.0%	0.0%	0.0%	1.8%	1.8%	0.0%	0.0%	0.0%
43	0.0%	0.0%	0.0%	3.5%	0.0%	0.0%	0.0%	0.0%
44	0.0%	1.8%	0.0%	0.0%	0.0%	0.0%	1.8%	0.0%
45	1.8%	0.0%	0.0%	0.0%	0.0%	1.8%	0.0%	0.0%
46	1.8%	0.0%	0.0%	0.0%	1.8%	0.0%	0.0%	0.0%
total	1.3%	0.8%	0.8%	0.8%	1.0%	0.6%	0.6%	0.5%

Summary of all performance errors induced by rTMS trains. Results are demonstrated as error rates per stimulation point and as the sum of all stimulation points for each language task.

#### Other error types

Altogether, phonological and semantic paraphasias, neologisms, and nominalization errors appeared very rarely (Figure 4a and b; Table 3). We therefore did not list errors in a detailed table. Differences between the utilized frequencies concerning other error types revealed no statistically significance (Table 3).

## Discussion

rTMS is a very promising technique for language mapping in preoperative planning. Despite some recently published convincing rTMS data [12,15], we are also aware of the present limitations of this technique that emerged, e.g. when performing repeated rTMS measurements or when comparing the results of rTMS with those of the current gold standard: direct cortical stimulation (DCS) during awake craniotomy [8,21]. Therefore, it is mandatory to improve the accuracy of rTMS in clinical applications, whereby delivering rTMS pulses appropriately might be one of the most important issues. The present study revealed differences in language impairment during stimulation with two distinct mapping frequencies, even though these frequencies only varied by a small amount. In the following, we discuss those distinctions and their relevance to the rTMS language-mapping protocol.

### Comparison of language impairment

To minimize variations that did not result from the distinct frequencies, we performed both investigations by the same observer. The video analysis was blinded to previous results and stimulated sites. Furthermore, we aimed to apply comparable stimulation intensities until the end of the duration of a burst in both mappings, so the stimulation at a repetition rate of 5 Hz and 10 pulses lasted 1.8 s, whereas at the higher repetition rate of 7 Hz and 10 pulses, it was only 1.29 s. In accordance with the current literature on the time course of brain activation during picture naming, word processing should be completed before our shorter stimulation duration of 1.29 s [22,23], and therefore variations should not be ascribed to different stimulation times. Yet, by applying the same number of pulses, the applied energy was also comparable in both groups. Although previous studies showed increased discomfort with higher frequencies [4], our results could not confirm these findings and therefore discomfort should not have influenced error rates.

However, our results varied significantly between groups. We suppose basic principles of rTMS played an important role in our observations. rTMS as neuropsychological research technique is able to disrupt language processes by creating a ‘virtual lesion’ , i.e. by inhibiting the underlying neural tissue. Yet there also are studies that describe rTMS-related facilitation of test performance. Those modulatory effects of rTMS on cortical excitability were reported to be frequency dependent. Several groups observed rTMS-induced naming or reading facilitation both in patients and healthy volunteers [24-28]. Those previous investigations vary in the used frequency, stimulation duration, stimulation intensity, and the location of applied rTMS, and deliver conflicting results. Nevertheless, a trend that higher frequencies showing facilitation effects seems likely. Since the aim of our investigation was not to examine modulatory effects of rTMS in detail, we used different stimulation parameters. We however observed a tendency of fewer errors when applying higher stimulation frequency, which might be due to facilitation effects in language processing during task performance.

On the other hand, the higher frequency tended to show a higher number of no response errors. A possible reason for our observation could rely on selective vulnerability of different language-related structures. No response errors are supposed to be a consequence of facial muscular activity (motor speech) [29], as well as of interference with language processing per se [1,30]. A previous repetitive TMS investigation in healthy subjects of Stewart et al. [30] reported that is seemed to be easier to resist nonmotoric than motoric disruption. In our study, stimulation sites that revealed higher no response error rates were mainly localized in mMFG, mPrG, and vPrG (Table 5), i.e., regions involved in speech-motor processing. These findings suggest that motoric language disruption might occur more often when applying higher frequencies.

Whereas in some error categories and language tasks, we observed distinct differences between the two applied frequencies, in certain error types and tasks we also obtained only minor variances when using one or the other frequency. Performance errors, for instance, but also phonological and semantic paraphasias, neologisms, and nominalization errors tend to be more frequent at 5 Hz (Figure 4a and b; Table 3). Nevertheless, in these error categories rTMS only evoked a small number of errors without significant differences between both frequencies. Furthermore, within all language tasks, the pseudoword reading task revealed low error rates in all error categories (Table 3) and thus no statistically significant differences during the two mapping sessions. The small number of language disturbances in this task is indicative that reading seems to be not that vulnerable to rTMS compared to naming tasks. In contrast to naming and generating tasks, reading demands no conceptually lemma selection [31] and therefore might involve comparatively less brain areas simultaneously.

### Practical implications

The present study showed a higher number of no response errors at a frequency of 7 Hz, whereas in all other error categories more disturbances of language processing could be found at a frequency of 5 Hz. This indicates that the language-mapping frequency could be tailored to which kind of error type you preferably want to provoke: the lower frequency, 5 Hz, seems to be more suitable for hesitation errors, performance errors, semantic and phonological paraphasias, neologisms and nominalization errors, whereas the higher frequency, 7 Hz, causes more no response errors.

### Limitations

All participants were healthy volunteers, so we had no opportunity to compare the results of rTMS to the current gold standard, DCS. On the other hand, with this study design, the present investigation allows us to examine the influence of the mapping frequency on the appearance of language disruption without the induced effects of cortical reorganization in case of brain lesions.

Furthermore, one of the basic limitations in rTMS studies is the fact that not all brain regions are accessible to stimulation due to their location at greater depths from the scalp or because of inacceptable pain. However, the lower frequency repetition rates used in this study already attempt to minimize pain during stimulation and thus to maximize the mapping area. As 5 Hz did not seem to cause less pain than 7 Hz, decreasing other parameters like stimulation intensity might be rather effective in reducing discomfort than continuing to decrease the frequency.

Further reasons for differences between the results of the first and the second investigation can be the spatial inconsistency of the investigated language function itself [32,33]. Therefore, the observed variation in language-related brain regions might not only appear as a result of the varying frequencies, but also due to changed functional organization of the human brain. Thus, we set a period of 13 to 15 days between the two sessions in order to prevent learning effects due to a long period in between but also reorganization by choosing thea shorter time period.

## Conclusion

This rTMS study questions if there is a discrepancy in the location, number, and type of errors when applying two different lower-frequency repetition rates in language mapping. Indeed, we observed in naming and generation tasks that with regard to all evoked language errors, a repetition rate of 5 Hz evoked a higher error rate than 7 Hz. Stimulation with the higher frequency, 7 Hz, however, provoked a higher number of total no response errors. It therefore might be beneficial to adjust the mapping frequency corresponding to the error type of interest in each individual mapping. Nevertheless, although both frequencies showed a well tolerable and efficient applicability, the rTMS language-mapping protocol still has to be refined, whereby we should also apply attention to other important parameters like stimulation onset, number of pulses, and stimulation intensity.

## Abbreviations

CPS: Cortical parcellation system
DCS: Bipolar direct cortical stimulation
nTMS: navigated transcranial magnetic stimulation
RMT: Resting motor threshold
rTMS: repetitive nTMS
VAS: Visual analogue scale
